# Ancient Schwannoma of the Submandibular Gland in a Patient With Acromegaly: A Case Report

**DOI:** 10.7759/cureus.98167

**Published:** 2025-11-30

**Authors:** Zerrin Ozergin Coskun, Muhammed Sadikzade, Recep Bedir

**Affiliations:** 1 Otolaryngology - Head and Neck Surgery, Recep Tayyip Erdoğan University Faculty of Medicine, Rize, TUR; 2 Medical Pathology, Recep Tayyip Erdoğan University Faculty of Medicine, Rize, TUR

**Keywords:** acromegaly, ancient schwannoma, igf-1, peripheral nerve sheath tumor, submandibular gland

## Abstract

Ancient schwannoma is a rare degenerative variant of schwannoma. Its occurrence in the submandibular gland is exceptionally uncommon, and, to the best of our knowledge, its coexistence with acromegaly has not been previously documented. A 22-year-old male patient with a history of acromegaly presented with a long-standing, painless swelling in the left submandibular region. Magnetic resonance imaging (MRI) revealed a well-defined, heterogeneous T2-hyperintense lesion with cystic and calcific components within the left submandibular gland. The patient underwent complete surgical excision of the affected gland. Histopathological examination demonstrated a spindle-cell neoplasm with nuclear palisading, cystic degeneration, and dystrophic calcifications, consistent with ancient schwannoma. Immunohistochemistry showed diffuse, strong S-100 protein positivity and a low Ki-67 proliferation index (2-3%), confirming the benign nature of the tumor. This case describes a rare coexistence of a submandibular ancient schwannoma and acromegaly in a young patient. Although a causal relationship between excess growth hormone (GH) or insulin-like growth factor 1 (IGF-1) and schwannoma development cannot be established from a single case, this association is hypothesis-generating and highlights the need for careful head and neck evaluation in patients with endocrine disorders. The patient had an uneventful postoperative course, and no recurrence was observed at six-month follow-up.

## Introduction

Schwannomas are benign neoplasms arising from Schwann cells of the peripheral nerve sheath. They most frequently occur in cranial nerves (especially the vestibular nerve), spinal nerve roots, and extremities, and exhibit a slow, indolent growth pattern [1]. A histological variant, known as ancient schwannoma, is characterized by degenerative features such as cystic degeneration, hemorrhage, nuclear atypia, and calcifications due to its long-standing nature [2,3].

Although schwannomas can arise throughout the body, their occurrence in the submandibular gland is extremely rare, with fewer than 20 well-documented cases reported in the literature [4,5]. The rarity of this localization often results in misdiagnosis or confusion with other salivary gland tumors.

Acromegaly, a systemic endocrine disorder caused by excessive growth hormone (GH) secretion, can predispose patients to both benign and malignant neoplasms. Elevated GH and insulin-like growth factor 1 (IGF-1) levels are known to stimulate cellular proliferation through mitogenic signaling pathways and have been implicated in increased overall tumor risk, although a direct causal link with schwannoma development has not been established [6,7]. Clinically, patients with acromegaly typically present with coarse facial features, frontal bossing, mandibular prognathism, enlarged hands and feet, and soft tissue thickening, which were evident in our patient. 

This case presents a rare intersection of two unusual conditions: a submandibular ancient schwannoma in a young male patient with a history of acromegaly. We aim to contribute to the existing literature by discussing the clinical presentation, diagnostic pathway, histopathological features, and the potential endocrine-oncologic association in this unique case.

## Case presentation

A 22-year-old male patient presented with a long-standing swelling in the left submandibular region, which he had first noticed approximately three years earlier. His medical history included a GH-secreting pituitary adenoma and acromegaly. Acromegaly had been diagnosed at the age of 18 years after he presented with progressive enlargement of the hands and feet, coarse facial features, frontal bossing, and mandibular prognathism.

He underwent transsphenoidal surgery for the pituitary adenoma one year after diagnosis. Postoperatively, GH and IGF-1 levels decreased but did not normalize completely, and he continued to show clinical features consistent with acromegaly. Therefore, long-acting somatostatin analogue therapy was initiated, together with testosterone replacement due to hypogonadism, and levothyroxine and prednisolone for postoperative hypopituitarism. There was no history of smoking or alcohol use, and no family history of tumors or endocrine disorders.

**Figure 1:**
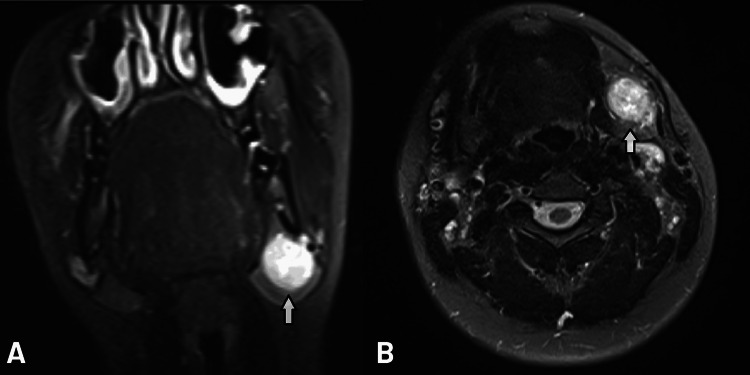
Preoperative magnetic resonance imaging (MRI) of the submandibular mass A: Coronal T2-weighted image shows the craniocaudal extent of the mass (arrow) and its anatomical relationship with the submandibular gland parenchyma and adjacent soft tissues. The predominantly hyperintense signal further supports the presence of cystic components. B: Axial T2-weighted image demonstrates a well-defined, hyperintense mass (arrow) in the left submandibular region. The lesion exhibits heterogeneous signal intensity, with internal areas of high signal suggestive of cystic degeneration and foci of low signal consistent with dystrophic calcifications.

Physical examination revealed a painless, firm, approximately 2 cm mass in the left submandibular region. Neck ultrasonography (USG) showed a 32 × 22 mm lobulated, hypoechoic nodular lesion with cystic spaces and echogenic septa within the left submandibular gland, with central vascularity on color Doppler ultrasonography (CDUS). Magnetic resonance imaging (MRI) revealed a 35 × 24 mm heterogeneously enhancing mass in the inferior part of the left submandibular gland. The coronal T2-weighted image demonstrated the craniocaudal extent of the mass and its relationship with the submandibular gland (Figure 1A). On T2-weighted axial images, the lesion appeared hyperintense with heterogeneous signal intensity, consistent with cystic degeneration and dystrophic calcifications (Figure 1B).

A Tru-cut biopsy result was consistent with a benign mesenchymal tumor. The biopsy demonstrated benign spindle-cell proliferation without mitotic activity or necrosis, consistent with a benign mesenchymal tumor.

**Figure 2:**
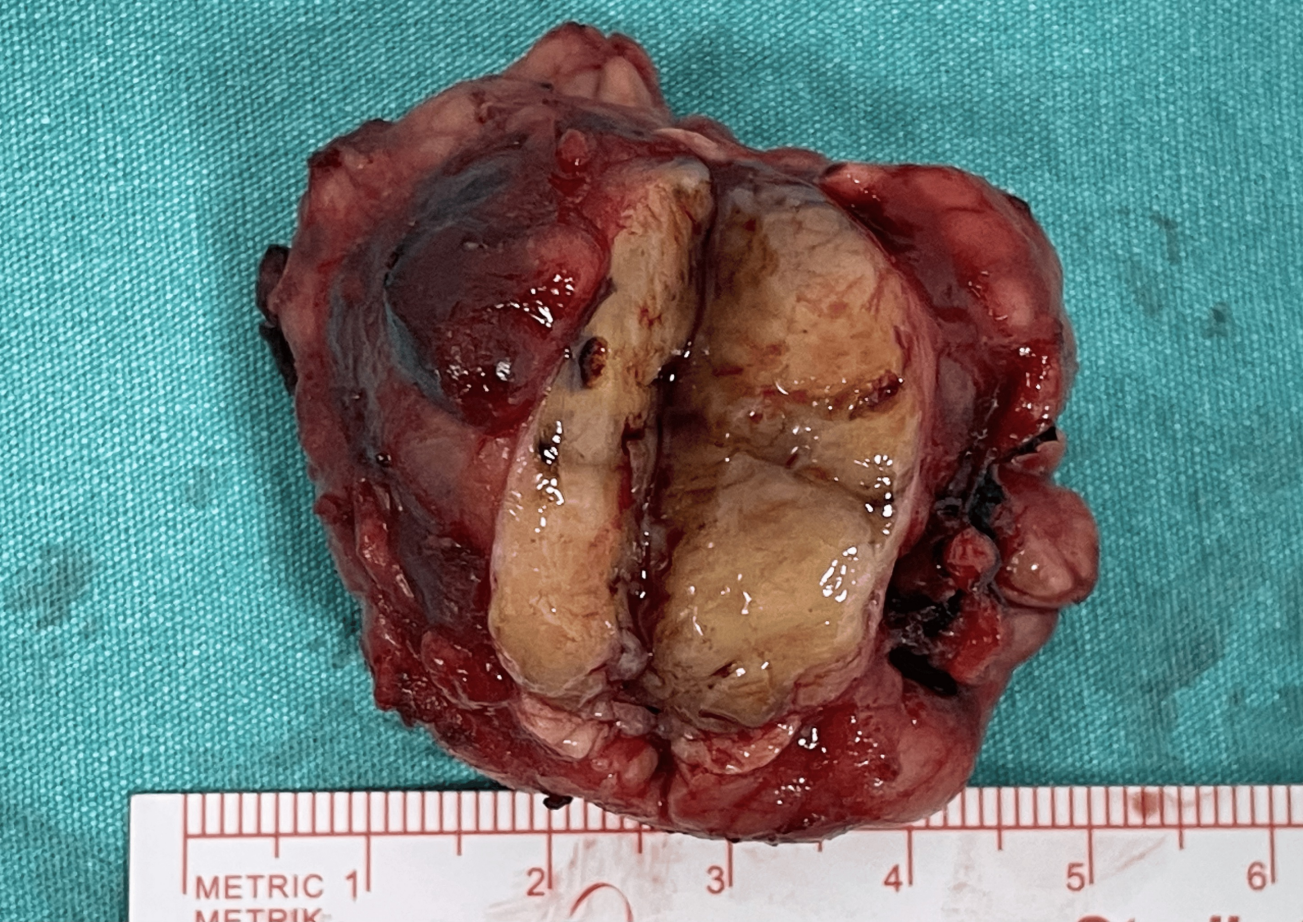
Gross pathological specimen of the excised submandibular gland The specimen shows a well-circumscribed, encapsulated mass measuring 3.5 cm in diameter. The cut surface is tan-white and exhibits a variegated appearance with prominent areas of cystic degeneration and focal, gritty calcifications.

The patient underwent surgery, and the left submandibular gland was completely excised. Although the lesion appeared well circumscribed, it was located within the parenchyma of the submandibular gland, and preoperative Tru-cut biopsy could not completely exclude a low-grade malignant process; therefore, an en bloc excision of the entire left submandibular gland containing the tumor was performed in accordance with standard salivary gland oncologic practice. Pathological examination confirmed the diagnosis of an ancient schwannoma. Gross examination of the excised specimen revealed a well-circumscribed, encapsulated mass measuring 3.5 cm in diameter. The cut surface of the tumor was tan-white in color with areas of cystic degeneration and focal calcifications (Figure 2).

**Figure 3:**
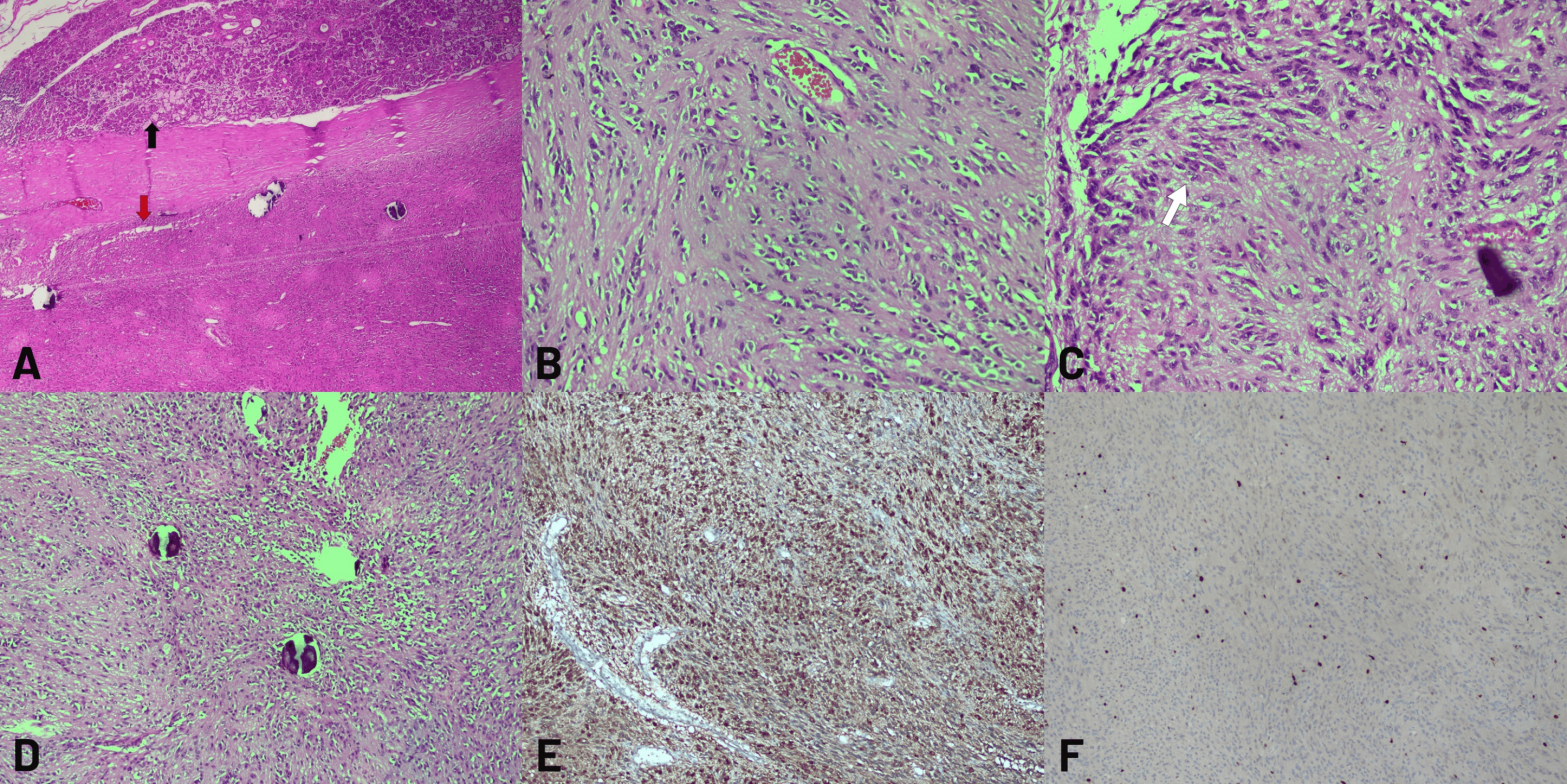
Histopathological features of the tumor A: An encapsulated spindle cell mesenchymal tumor (red arrow) is observed adjacent to the salivary gland (black arrow) (H&E x40). B: The tumor consists of proliferating spindle cells (H&E x100). C: Nuclear palisading (white arrow) is observed in the tumor (H&E x200). D: Dystrophic calcification is observed in the tumor (H&E x100). E: Diffuse positive staining with S-100 is observed in the tumor (x100). F: Low Ki-67 proliferation is observed in the tumor (x100).

Microscopic examination revealed a spindle cell mesenchymal tumor with characteristic features of an ancient schwannoma. The tumor was encapsulated and adjacent to the salivary gland tissue. It was composed of proliferating spindle cells arranged in fascicles. Nuclear palisading, a hallmark feature of schwannomas, was observed in focal areas. Additionally, dystrophic calcifications, indicative of long-standing degenerative changes, were noted within the tumor stroma (Figures 3A-3D).

Immunohistochemical staining was performed to confirm the diagnosis. The tumor cells exhibited diffuse and strong positivity for S-100 protein (Figure 3E), supporting their Schwann cell origin. The Ki-67 proliferation index was low (2-3%) (Figure 3F), consistent with the benign nature of the tumor. These findings collectively confirmed the diagnosis of ancient schwannoma.

The postoperative period was uneventful, and the patient was discharged without complications. No recurrence was observed during the six-month follow-up period.

## Discussion

Ancient schwannomas are histologically benign tumors, but their degenerative features, such as nuclear atypia, cystic changes, and calcifications, can raise concerns for malignancy both radiologically and histologically [1-3]. This can pose diagnostic challenges, especially when they present in rare locations, such as the submandibular gland, where they mimic more common salivary gland neoplasms [4,5].

Clinical and histopathological differentials were considered prior to establishing the diagnosis. Clinically, the submandibular mass raised differential diagnoses including pleomorphic adenoma, myoepithelioma, chronic sialadenitis, and reactive or neoplastic lymphadenopathy. Histopathologically, spindle-cell lesions, including neurofibroma, low-grade malignant peripheral nerve sheath tumor (MPNST), spindle-cell myoepithelioma, and solitary fibrous tumor, were evaluated. Diffuse and strong S-100 positivity, absence of atypical mitotic activity, well-formed nuclear palisading, and a very low Ki-67 index favored benign schwannoma over these entities. These features, together with encapsulation and degenerative changes, supported the diagnosis of ancient schwannoma.

Endocrine disturbances are not commonly associated with schwannoma development, yet isolated reports suggest that hormonal alterations may influence tumor growth. Saito et al. described a giant cauda equina schwannoma in a patient with marked dural ectasia and discussed the potential contribution of systemic factors to connective tissue remodeling and nerve sheath changes [8]. Although the available evidence is very limited and does not establish causality, such reports raise the possibility that endocrine dysregulation may modify Schwann cell behavior. In our case, the coexistence of acromegaly and an ancient schwannoma is intriguing; however, given the rarity of both conditions and the absence of a well-established biological mechanism linking GH/IGF-1 excess to schwannoma formation, a coincidental association cannot be excluded. Moreover, the exact duration and biochemical control of our patient’s acromegaly were not fully documented, which further limits any causal inference. Therefore, this case should be regarded as hypothesis-generating and suggests that the potential influence of GH/IGF-1 on peripheral nerve sheath tumors merits further investigation [6,7,9].

Only a limited number of submandibular ancient schwannoma cases have been reported [4,5]. Park et al. reported seven submandibular schwannomas, of which only two exhibited definitive ancient features [5]. In a literature review by Ho et al., ancient variants accounted for less than 5% of all submandibular gland tumors [4]. Our case adds to this sparse literature and is further distinguished by the presence of a systemic endocrine condition, acromegaly, which has not been previously reported in association with submandibular ancient schwannomas.

Acromegaly, characterized by excess GH and subsequent IGF-1 elevation, is known to increase the risk of several neoplasms [6,9]. Although peripheral nerve sheath tumors are rarely reported in this context, the mitogenic properties of IGF-1 may contribute to schwannoma pathogenesis. Jenkins and Besser have previously suggested a potential oncogenic link between elevated IGF-1 and tumor proliferation [7].

Imaging plays a crucial role in preoperative planning. In our case, MRI revealed a hyperintense, heterogeneous lesion on T2-weighted images with characteristics suggestive of cystic degeneration and calcification, hallmarks of an ancient schwannoma [3]. While imaging is informative, definitive diagnosis requires histopathological and immunohistochemical examination. The tumor in our patient showed classic features and was strongly positive for S-100 protein, with a low Ki-67 index, supporting the benign diagnosis.

This case illustrates the diagnostic benefit of a multidisciplinary approach. Our patient underwent complete surgical excision with an uneventful recovery and no recurrence during follow-up, consistent with literature reports stating that complete resection offers an excellent prognosis [5,9,10].

## Conclusions

This is the first report to describe a submandibular ancient schwannoma in a patient with acromegaly. This coexistence is hypothesis-generating and raises the question of whether chronic GH/IGF-1 excess might influence nerve sheath tumor biology, although no causal relationship can be inferred from a single case. Comprehensive histopathological evaluation is crucial for a definitive diagnosis, and a multidisciplinary management strategy ensures optimal patient outcomes.
